# Dietary yeast culture improves growth performance and nutrient utilization while reducing odor-causing compounds and modulating cecal microbiota in broilers

**DOI:** 10.3389/fvets.2026.1891643

**Published:** 2026-08-11

**Authors:** Dongliang Wang, Xu Wang, Xiaoxue Yue, Qiwei Song, Xinping Yan, Zhikui Wang, Yingying Fu, Xiulei Cai

**Affiliations:** College of Veterinary Medicine, Qingdao Agricultural University, Qingdao, China

**Keywords:** Arbor Acres broilers, carcass traits, cecal microbiota, growth performance, meat quality, yeast culture

## Abstract

Dietary yeast culture supplementation has been proposed as an effective strategy to enhance production efficiency and intestinal health in poultry. This study evaluated the effects of yeast culture (YC) on growth performance, nutrient utilization, odor-related metabolites, and cecal microbiota in broilers. A total of 960 one-day-old Arbor Acres broilers were randomly assigned to three dietary treatments with 16 replicates per treatment and 20 birds per replicate. Compared with the control group, YC supplementation increased average daily gain (ADG) and average daily feed intake (ADFI), and improved the feed conversion ratio (F/G) during the early and overall growth periods (*p* < 0.05). Nutrient utilization was significantly enhanced, as reflected by increased crude fat (CF), crude ash (Ash), and crude protein (CP) digestibility across different growth phases (*p* < 0.05). In addition, YC supplementation reduced the concentrations of indole and skatole (*p* < 0.05), two major odor-causing metabolites, and decreased muscle triglyceride (TG) and total cholesterol (TC) levels (*p* < 0.05). Microbiota analysis showed that YC supplementation significantly increased the relative abundance of beneficial genera, including Lactobacillus and Barnesiella. In conclusion, dietary YC improved growth performance and nutrient utilization, reduced odor-related metabolites, and selectively altered specific bacterial genera, particularly Lactobacillus and Barnesiella. This study provides evidence for the use of YC as a beneficial feed additive for improving production performance and reducing odor-related compounds in broilers.

## 1 Introduction

Antibiotics are widely used in animal husbandry to enhance productivity, improve disease resistance, and act as growth promoters or prophylactic agents (1). Despite their notable contributions to improving animal production performance, the use of antibiotics is accompanied by significant drawbacks. Prolonged administration of antibiotics can lead to the emergence of drug-resistant bacterial strains, posing a substantial threat to global public health (2, 3). The overuse of antibiotics is a primary driver of the development of drug-resistant genes in bacteria. This phenomenon can facilitate the spread of resistant bacteria, which may impact human health. In addition, antibiotics affect the gut microbiota of animals. Besides their bactericidal effects, antibiotics may disrupt beneficial microbiota, thereby impairing the animal’s immune system, digestion, absorption, and overall health (4, 5). Moreover, antibiotic residues in animal products may enter the human food chain, raising potential health concerns. The improper use of antibiotics can adversely affect both animal and human health.

In response to the global concern regarding antimicrobial resistance, many countries have implemented policies restricting or banning the use of antibiotics in animal production systems. For instance, the European Union enforced a complete ban on antibiotic growth promoters in 2006 (6). Similarly, countries such as China are enforcing stricter regulations on antibiotic use (7). Consequently, identifying safe, sustainable, and effective alternatives to antibiotics has become a pivotal research objective. The ban on antibiotics aims not only to mitigate antibiotic resistance but also to reduce environmental contamination. Novel additives, including probiotics and prebiotics, can enhance animal immunity, promote the growth of beneficial bacteria, and improve intestinal health (8, 9). Phages have antibacterial, anti-inflammatory, and antiviral properties (10). Herbs have been traditionally used for green prevention and control, showing potent antimicrobial, antiviral, and immune-modulating effects (11). These alternatives not only reduce antibiotic usage but also promote animal health and enhance production efficiency.

Yeast culture (YC), used as feed additives, are rich in proteins, amino acids, vitamins, and nucleic acids. The benefits of yeast and its metabolites for animal health and immune function are well documented, having been extensively studied and applied in animal husbandry practices. In dairy and beef cattle, goats, and sheep, YC has been reported to enhance rumen health and milk production (12, 13). In aquaculture, YC has been shown to improve growth performance and disease resistance in fish and shrimp (14). Additionally, YC has been reported to promote the growth of nursery pigs (15). In poultry, YC has been shown to improve intestinal microecological balance, contribute to improved intestinal barrier function, reduce harmful bacterial proliferation, prevent diseases, and boost immunity (16). Emerging evidence suggests that the gut microbiota plays an important role in nutrient utilization, intestinal homeostasis, and growth performance in poultry, and the beneficial effects of YC may be partially associated with its interactions with the intestinal microbial ecosystem. YC is therefore considered a promising feed additive due to its beneficial effects on intestinal health, immune function, and growth performance in poultry. Collectively, these findings indicate that YC may improve broiler growth performance and intestinal health, potentially through microbiota-mediated mechanisms, providing a basis for further investigation of YC–gut microbiota interactions in broiler production systems.

## 2 Materials and methods

### 2.1 Animal ethics statement

Animal ethics: In this study, all animal procedures were performed following the experimental protocol approved by the Qingdao Agricultural University Animal Care and Use Committee (approval number: QAU2023021102).

### 2.2 Preparation of YC product

Preparation of YC: The yeast culture (YC) used in this study was provided by Diamond V. (Shenzhen, China). Fermented by *Saccharomyces cerevisiae*, the product has a guaranteed analysis as follows: crude protein ≥19%, crude fat ≥1.0%, crude fiber ≤18.5%, crude ash ≤12.0%, moisture ≤11.0%, mannan ≥2.0%.

### 2.3 Experimental design

A total of 960 1-day-old Arbor Acres broiler chicks with similar body weights (46.87 ± 0.79 g) were used in a 42-day feeding trial. The animals were randomly assigned to 3 groups, each consisting of 16 replicates with 20 chicks per replicate pen. All the chickens were reared in the experimental poultry house at Qingdao Agricultural University under standardized management conditions. The experiment was conducted in three phases: phase 1 (days 1–10), phase 2 (days 11–20), and phase 3 (days 21–42). The control group (CON) was fed a basal diet, dietary treatments were as follows: CON group (basic diet), T1 group (YC was supplemented in a stepwise manner, with 0.625 kg/t for days 1–10 and 0.3125 kg/t for days 11–42), T2 group (YC was added to the basal diet at 0.625 kg/t throughout). The basal diet was formulated as a corn–soybean meal–miscellaneous meal according to the Chinese Chicken Feeding Standard (NY/T 33-2004). The composition and nutritional levels of the basal diet are presented in Table 1. The chickens were immunized on day 7 with a live Newcastle disease and infectious bronchitis vaccine via one drop in the eye, at 14 days with an infectious bursal disease vaccine via one drop in the eye, and at 21 days with a live Newcastle disease + infectious bronchitis vaccine via one drop in the nose. The poultry house was cleaned regularly and disinfected weekly. The temperature was initially maintained at 33 °C ± 1 °C for the first 3 days and was then gradually decreased by 0.3 °C per day until reaching 24 °C, which was maintained throughout the experiment. The humidity was maintained at approximately 60%. Artificial fluorescent lights were used continuously. The broilers had free access to feed and water in double cages (120 cm × 80 cm × 60 cm) equipped with troughs and two nipple drinkers.

**Table 1:** Ingredient and nutrient composition of the experimental diets.

Ingredient, %	Basal diet		
	0–10 d	11–20 d	21–42 d
Corn	58.801	62.463	64.403
Soybean meal	33.743	28.389	24.375
Cottonseed meal, 47%	2.5	3	4
Soy oil	1.248	2.664	3.973
Limestone powder	1.128	1.121	1.102
Dicalcium phosphate, anhydrous	1.058	0.783	0.673
DL-Met	0.347	0.333	0.305
L-Lys HCL, 98%	0.273	0.305	0.288
Salt (NaCl)	0.221	0.214	0.222
Sodium bicarbonate	0.197	0.212	0.204
Threonine	0.177	0.164	0.129
Mineral premix^a^	0.1	0.1	0.1
Choline chloride	0.06	0.063	0.057
Val	0.048	0.065	0.054
Ile	0.029	0.054	0.05
Vitamin premix^b^	0.03	0.03	0.025
Emulsifier	0.03	0.03	0.03
Phytase 20,000	0.01	0.01	0.01
Total	100	100	100
Nutritional parameters			
ME, Mcal/kg	2.9	3.01	3.1
Crude protein, %	22.51	20.52	19.19
Crude fat, %		5.3	6.6
Crude fiber, %	2.72	2.68	2.69
Crude ash, %	4.02	3.84	3.71
Ca, %	0.85	0.75	0.7
Total P, %	0.65	0.56	0.53
Available P, %	0.35	0.28	0.25

a Mineral premix contained the following per kilogram of diet: Cu 6 mg, Fe 50 mg, Mn 90 mg, I 0.50 mg, Se 0.30 mg, Zn 70 mg.

b Vitamin premix contained the following per kilogram of diet: VA 10000 IU, VB_1_ 2 mg, VB_2_ 7 mg, VD_3_ 2,750 IU, VE 17 mg, VK_3_ 2 mg, D-pantothenic acid 10 mg, nicotinic acid 40 mg, folic acid 0.25 mg.

### 2.4 Growth performance, nutrient digestibility and excreta metabolites

Each group consisted of 16 pens, with each pen housing 20 birds. The pen served as the experimental unit for measuring growth performance parameters. Body weight and feed intake were recorded on days 0, 11, 21, and 42. From these measurements, the average body weight (ABW), average daily feed intake (ADFI), average daily gain (ADG), and feed/gain ratio (F/G) were calculated. Nutrient digestibility and analyses of excreta metabolites, such as indole and skatole, were also evaluated.

### 2.5 Sample collection

At the end of the experiment, two chickens per replicate were randomly selected based on body weight, ensuring values were close to the replicate mean. The parameters of live weight, dressed weight, eviscerated weight, half-eviscerated weight with giblet, pectoral muscle weight, leg muscle weight, and abdominal fat weight were calculated. The slaughter method was performed according to the Performance Terminology and Measurements for Poultry (NY/T 823-2020) to determine the dressed percentage (DP), percentage of eviscerated carcass (PEC), percentage of partly eviscerated carcass (PPEC), percentage of breast muscle (PBM), percentage of drumstick muscle (PDM), and percentage of abdominal fat (PAF).

### 2.6 Fecal nutrient utilization determination

Excreta samples were collected from each pen on days 7–10, 17–20, and 38–41. Each 100 g of excreta was treated with 10% hydrochloric acid and stored at −20 °C until nutrient utilization analysis. Simultaneously, feed samples were collected from each group for analysis. Nutrient utilization was assessed in terms of dry matter (DM), crude ash (Ash), crude protein (CP), and crude fat (CF). Acid-insoluble ash (AIA) was used as an internal marker and determined in both feed and excreta samples. Acid-insoluble ash (GB/T 23742-2009), DM (GB/T 6435-2014), Ash (GB/T 6438-2007), CP (GB/T 6432-2018), and CF (GB/T 6433-2006) were estimated according to standardized analytical procedures. The following formula was employed to calculate the gross total utilization of nutrients: Nutrient utilization (%) = [1 − (A1/A2 × F2/F1)] × 100%, where A1 = acid insoluble ash in feed (%), A2 = acid insoluble ash in excreta (%), F1 = nutrient in feed (%), and F2 = nutrient in excreta (%).

### 2.7 Fecal foul odor compound determination

Fresh excreta samples were collected from each pen on days 26, 27, and 28 and stored at −20 °C until the analysis of skatole and indole. High-performance liquid chromatography (HPLC) was used (Agilent 1100 model, Agilent Technologies, Santa Clara, CA). For this procedure, 2 g of broiler fecal sample was placed into a 10 mL tissue grinder. Subsequently, 4 mL of distilled water was mixed for 1 min, 1 mL of the mixture was taken, and 2 mL of chromatography-grade methanol was added to it. The mixture was vortexed and placed at −20 °C for 30 min to accelerate the precipitation of particulate matter. The sample was centrifuged at 3,000 × *g* for 10 min. Later, 1 mL of the clear liquid was transferred to a 1.5 mL microtube. The tube was centrifuged at 15,000 × *g* for another 30 min, and the supernatant was transferred to a new microtube. The supernatant was aspirated using a 1 mL disposable syringe and filtered through a 0.22 μm filter membrane before being injected for analysis. The acetonitrile and methanol used for analysis were pure reagents obtained from Sinopharm Chemical Reagent Co. Ltd. (Shanghai, China). The indole and skatole standards used for analysis were purchased from Shanghai Yuanye Bio-Technology Co. Ltd. (Shanghai, China). The chromatographic column was a DiKMA Platisil C18 (250 × 4.6 mm, 5 μm). The other parameters were as follows: mobile phase: acetonitrile: water = 60: 40; flow rate: 1.0 mL/min; column temperature: 30 °C; fluorescence detector: excitation wavelength, 263 nm; emission wavelength, 358 nm; sample size: 20 μL.

### 2.8 Muscle nutrient analysis

At 42 days, two chickens per replicate were randomly selected from each group based on their weights, which were close to the average. Breast muscle samples were subsequently collected for nutrient composition analysis. Nutrients in muscle crude protein (CP) and crude fat (CF) were determined following the standards GB 5009.5-2016 (China) and GB 5009.6-2016 (China), respectively. Inosine monophosphate (IMP), triglyceride (TG), and total cholesterol (TC) were assayed using kits from Shanghai Enzyme Linkage Biotechnology Co. Ltd. (Shanghai, China). The catalog numbers are: Y1042771, YJ495832 and YJ594030. The intra-assay and inter-assay coefficients of variation of the ELISA kits were <15%.

### 2.9 Cecal microbiota analysis

At 28 days, six broiler chickens with similar body weight were selected from each group. Cecal contents were collected by scraping the cecum with a sterile slide. The samples were preserved in liquid nitrogen in 2 mL microtubes and sent to Shanghai Majorbio Bio-Pharm Technology Co., Ltd. for 16S rRNA sequencing of cecal microbiota (Shanghai, China). The V3–V4 hypervariable regions were sequenced on the Illumina NovaSeq platform via paired-end reads. Following quality filtering, the data were subjected to operational taxonomic unit (OTU) clustering and taxonomic classification. Representative sequences from each OTU cluster were taxonomically annotated, enabling the determination of microbial identities and the generation of species abundance profiles. For slaughter performance and muscle nutrient composition, the individual bird was considered the experimental unit, with two birds sampled per replicate and averaged before statistical analysis. For cecal microbiota analysis, one bird per replicate was selected (*n* = 6 per group), and each sample was treated as an independent experimental unit.

### 2.10 Statistical analysis

The data were analyzed using SPSS statistical software (version 22.0; SPSS Inc., Chicago, IL, USA). Growth performance data were analyzed using the pen as the experimental unit (*n* = 16 pens per treatment), whereas slaughter performance and muscle composition data were analyzed based on the mean values per replicate. Cecal microbiota data were analyzed at the individual bird level (*n* = 6 per group), while nutrient digestibility and indole/skatole analyses were performed using the pen as the experimental unit. Growth performance variables from different growth phases (0–10, 11–20, and 21–42 days) were analyzed separately. One-way analysis of variance (ANOVA) was used to evaluate differences among dietary treatments within each phase. When significant effects were detected, Tukey’s multiple comparison test was applied for *post hoc* comparisons. A probability value of *p* < 0.05 was considered statistically significant.

## 3 Results

### 3.1 Effect of yeast culture on growth performance of broilers

The effects of YC supplementation on the growth performance of broilers are presented in Table 2. The results showed that compared with the CON group, ADG was significantly increased in the T1 and T2 groups at 21 days (*p* < 0.05). Moreover, the ADG of the T1 group during the 0–42 days period was significantly higher compared to the CON group (*p* < 0.05). The ADFI during the 0–42 days period was significantly higher in the T1 and T2 groups compared to the CON group (*p* < 0.05). Furthermore, the F/G at 21 days was significantly lower in the T1 group compared to the CON group (*p* < 0.05). However, there was no significant difference in ABW compared to the CON group (*p* > 0.05).

**Table 2:** Effects of supplemental yeast culture in broiler diets on growth performance.

Item	CON	T1	T2	*p*-value
ABW (g)				
0 day	46.82 ± 0.61	46.93 ± 1.19	47.21 ± 0.86	0.583
11 days	212.57 ± 24.66	209.93 ± 15.48	217.89 ± 11.68	0.546
21 days	593.41 ± 45.68	612.02 ± 33.80	595.70 ± 29.95	0.419
42 days	2224.54 ± 102.73	2283.33 ± 98.52	2279.08 ± 151.92	0.453
ADG (g/d)				
11 days	16.57 ± 2.47	16.30 ± 1.51	17.07 ± 1.16	0.568
21 days	37.92 ± 1.52^b^	40.71 ± 1.71^a^	39.53 ± 1.56^a^	0.001
42 days	59.97 ± 3.52	61.12 ± 3.25	60.65 ± 4.24	0.777
0–42 days	43.10 ± 1.43^b^	45.48 ± 2.00^a^	44.83 ± 2.52	0.029
ADFI (g/d)				
11 days	21.90 ± 2.82	21.93 ± 1.54	22.73 ± 1.39	0.514
21 days	57.43 ± 2.79	59.21 ± 2.29	58.30 ± 2.05	0.204
42 days	119.95 ± 4.69	122.03 ± 3.70	121.35 ± 5.59	0.582
0–42 days	79.96 ± 1.85^b^	83.30 ± 2.27^a^	82.62 ± 3.35^a^	0.012
F/G				
11 days	1.34 ± 0.06	1.35 ± 0.06	1.33 ± 0.05	0.780
21 days	1.52 ± 0.07^a^	1.45 ± 0.04^b^	1.47 ± 0.03	0.009
42 days	2.00 ± 0.68	2.00 ± 0.61	1.99 ± 0.85	0.954
0–42 days	1.85 ± 0.03	1.83 ± 0.05	1.84 ± 0.43	0.514

ABW, average body weight; ADG, average daily gain; ADFI, average daily feed intake; F/G, feed/gain.

Values within the same row with different lowercase superscript letters are significantly different (*p* < 0.05).

### 3.2 Effect of yeast culture on slaughter performance of broilers

The effects of supplemental YC on the slaughter performance of broilers are presented in Table 3. The PBM was significantly higher in the T2 group than in the CON group (*p* < 0.05). Nonetheless, the effects of supplemental YC on PDM, PPEC, PEC, and DP tended to increase compared with the CON group, whereas PAF displayed a downward trend compared with the CON group (*p* > 0.05).

**Table 3:** Effects of supplemental yeast culture on the slaughter performance of broilers.

Item	CON	T1	T2	*p*-value
PBM/%	23.39 ± 0.69^b^	23.50 ± 1.07^b^	24.93 ± 1.60^a^	0.018
PDM/%	22.58 ± 1.20	22.76 ± 0.98	23.60 ± 1.59	0.208
PPEC/%	79.41 ± 1.83	79.93 ± 2.19	79.55 ± 1.97	0.824
PEC/%	66.56 ± 2.21	67.60 ± 1.27	67.09 ± 1.41	0.356
PAF/%	1.51 ± 0.32	1.45 ± 0.25	1.46 ± 0.27	0.891
DP/%	88.06 ± 1.19	88.22 ± 1.11	88.29 ± 1.03	0.903

PBM, percentage of breast muscle; PDM, percentage of drumstick muscle; PPEC, percentage of partially eviscerated carcass; PEC, percentage of eviscerated carcass; PAF, percentage of abdominal fat; DP, dressed percentage.

Values within the same row with different lowercase superscript letters are significantly different (*p* < 0.05).

### 3.3 Effect of yeast culture on nutrient digestibility of broilers

The effects of YC on the apparent metabolic rates of nutrients in broilers are listed in Table 4. The CF contents showed significant differences during the 1st phase, with the T1 and T2 groups having significantly higher levels than the CON group (*p* < 0.05). In the 2nd phase, the Ash and CF contents in the T2 group were significantly higher than those in the CON group (*p* < 0.05). In the 3rd phase, the CP contents in the T1 and T2 groups were significantly higher than those in the CON group (*p* < 0.05).

**Table 4:** Effects of yeast culture on the apparent metabolic rates of nutrients in broilers.

Item	CON	T1	T2	*p*-value
Phase 1 (0–10 days)				
DM/%	81.81 ± 4.29	84.75 ± 4.59	87.17 ± 4.84	0.162
Ash/%	36.73 ± 8.73	42.47 ± 3.64	44.43 ± 8.88	0.214
CP/%	49.81 ± 4.84	50.90 ± 4.41	50.26 ± 6.20	0.935
CF/%	62.26 ± 5.59^b^	78.75 ± 7.81^a^	73.88 ± 5.92^a^	0.002
Phase 2 (11–20 days)				
DM/%	80.17 ± 7.01	86.40 ± 2.27	84.32 ± 7.97	0.247
Ash/%	32.97 ± 6.82^b^	39.63 ± 6.36	42.53 ± 2.91^a^	0.029
CP/%	50.37 ± 6.79	54.31 ± 4.18	57.06 ± 2.66	0.087
CF/%	76.21 ± 3.93^b^	78.67 ± 3.29	82.19 ± 2.54^a^	0.022
Phase 3 (21–42 days)				
DM/%	77.51 ± 9.99	86.28 ± 3.44	83.29 ± 5.65	0.116
Ash/%	43.20 ± 7.02	53.82 ± 6.44	52.02 ± 9.27	0.065
CP/%	46.75 ± 2.11^b^	52.92 ± 4.07^a^	56.03 ± 6.73^a^	0.012
CF/%	58.78 ± 7.47	61.64 ± 7.04	61.23 ± 4.91	0.730

DM, dry matter; Ash, crude ash; CP, crude protein; CF, crude fat. The pen was considered the experimental unit (*n* = 16 pens per treatment).

Values within the same row with different lowercase superscript letters are significantly different (*p* < 0.05).

### 3.4 Effect of yeast culture on excreta odor compounds of broilers

The effects of YC supplementation on the contents of the main odor-causing compounds in the excreta are presented in Table 5. The addition of YC significantly reduced the fecal indole and skatole levels compared with the CON group (*p* < 0.05).

**Table 5:** Effects of supplemental yeast culture on indole and skatole concentrations of broilers.

Item	CON	T1	T2	*p*-value
Indole (μg/g)	51.05 ± 1.69^a^	47.64 ± 1.37^b^	47.73 ± 1.16^b^	0.044
Skatole (μg/g)	28.23 ± 1.07^a^	26.53 ± 0.19^b^	26.88 ± 0.29^b^	0.040

The pen was considered the experimental unit (*n* = 16 pens per treatment).

Values within the same row with different lowercase superscript letters are significantly different (*p* < 0.05).

### 3.5 Effect of yeast culture on muscle nutrients compounds of broilers

As shown in Table 6, the TG and TC levels differed significantly among treatments, with levels in the T2 group being significantly lower than those in the CON group (*p* < 0.05). Compared with the CON group, there was no significant difference in IMP levels (*p* > 0.05). YC supplementation did not significantly affect the CP and CF contents in the muscle (*p* > 0.05).

**Table 6:** Effects of supplemental yeast culture on the nutrient contents of broilers.

Item	CON	T1	T2	*p*-value
IMP (μg/mL)	165.10 ± 13.88	163.62 ± 10.96	173.58 ± 14.18	0.387
TG (μg/mL)	6.37 ± 0.31^a^	6.12 ± 0.32^a^	5.61 ± 0.44^b^	0.007
TC (μg/mL)	9.87 ± 0.91^a^	9.79 ± 1.02^a^	9.12 ± 1.42^b^	< 0.01
CP/%	24.74 ± 0.80	26.10 ± 1.29	24.83 ± 1.44	0.128
CF/%	1.70 ± 0.20	1.79 ± 0.15	2.03 ± 0.45	0.183

IMP, inosine monophosphate; TG, triglyceride; TC, total cholesterol; CP, crude protein; CF, crude fat.

Values within the same row with different lowercase superscript letters are significantly different (*p* < 0.05).

### 3.6 Effect of yeast culture on microbiota composition of broilers

**Figure 1:**
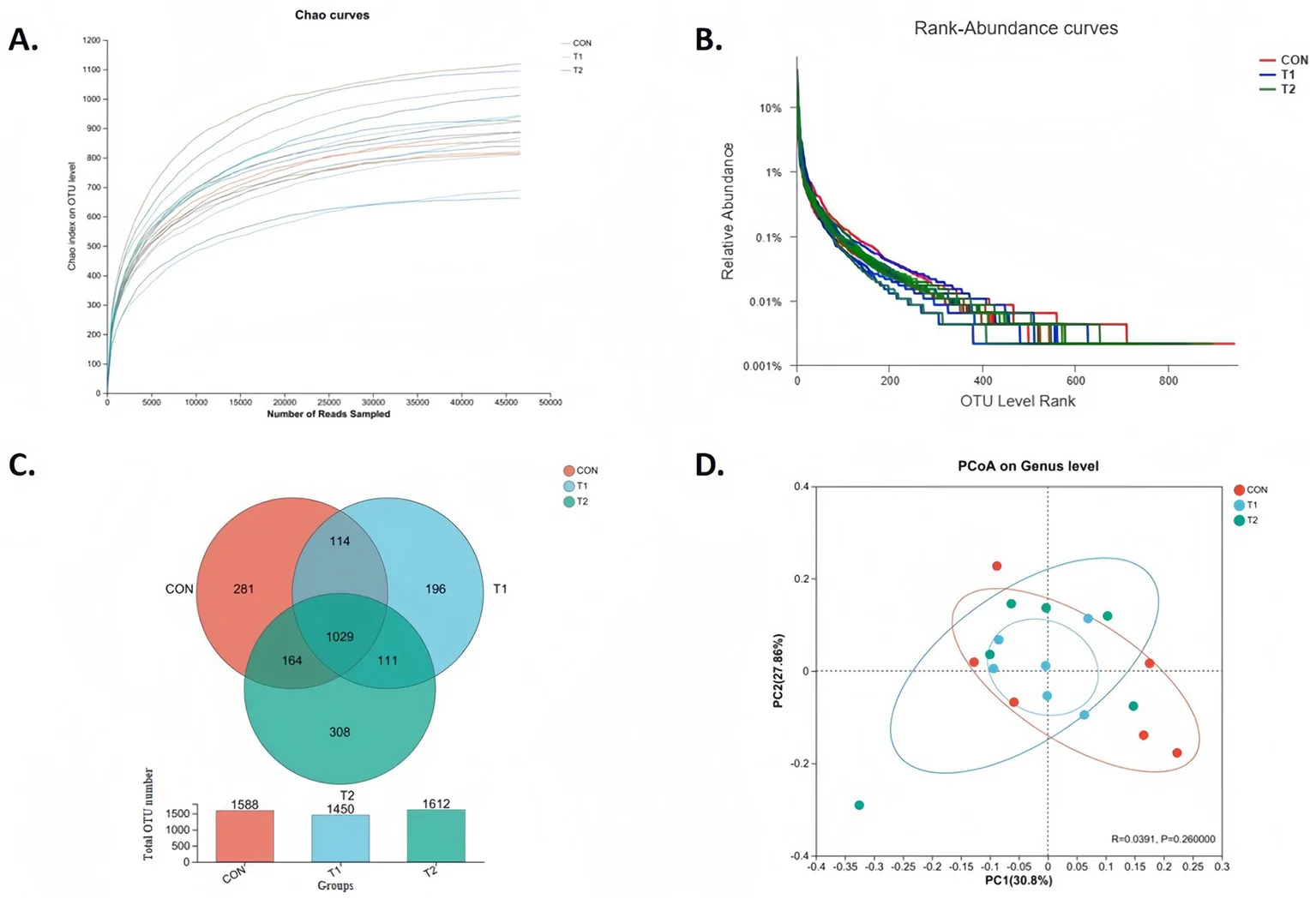
Microbial diversity analysis of cecum in broiler chickens. **(A)** Rarefaction curve; **(B)** rank abundance curve; **(C)** Venn diagram; **(D)** beta diversity index.

A total of 1,472,652 valid sequences were obtained from 18 samples. As depicted in Figure 1A, as the number of sequenced sequences increased, the rarefaction curve flattened and reached a plateau, indicating that the sequencing depth was sufficient to capture the majority of microbial diversity in the samples. The flattening of the OTU rank abundance curve further indicated relatively high species richness and evenness in the cecal microbiota across samples (Figure 1B). Valid sequences with 97% similarity were clustered into OTUs, with a total of 4,650 OTUs clustered together in the three groups and 1,029 OTUs shared among them. The percentages of group-specific OTUs were 17.70, 13.52, and 19.11%, respectively (Figure 1C). As shown in Table 7, significant differences were not observed in the Shannon, Simpson, Ace, or Chao1 indexes among the various groups (*p* > 0.05). The coverage was >0.99. The *β* diversity obtained from the principal coordinates analysis (PCoA) plot of the weighted UniFrac analysis of the cecal microbiota is presented (Figure 1D). In the PCoA plot, PC1 and PC2 explained 30.8 and 27.86% of the total variation, respectively. Samples exhibited partial clustering with considerable overlap among groups, indicating no distinct separation in overall cecal microbial community structure among treatments (*p* > 0.05).

**Table 7:** Effects of supplemental yeast culture on the alpha diversity of gut microbiota in broilers.

Item	CON	T1	T2	*p*-value
Shannon index	2.80 ± 0.32	2.88 ± 0.22	2.81 ± 0.18	0.856
Simpson index	0.15 ± 0.06	0.11 ± 0.03	0.13 ± 0.03	0.380
Ace index	118.95 ± 8.14	119.29 ± 4.04	120.59 ± 10.59	0.934
Chao1 index	118.33 ± 7.40	122.02 ± 1.38	120.18 ± 11.18	0.718
Coverage	0.99 ± 0.00004	0.99 ± 0.00004	0.99 ± 0.00011	0.567

Values within the same row with different lowercase superscript letters are significantly different (*p* < 0.05).

### 3.7 Taxonomy summary

**Figure 2:**
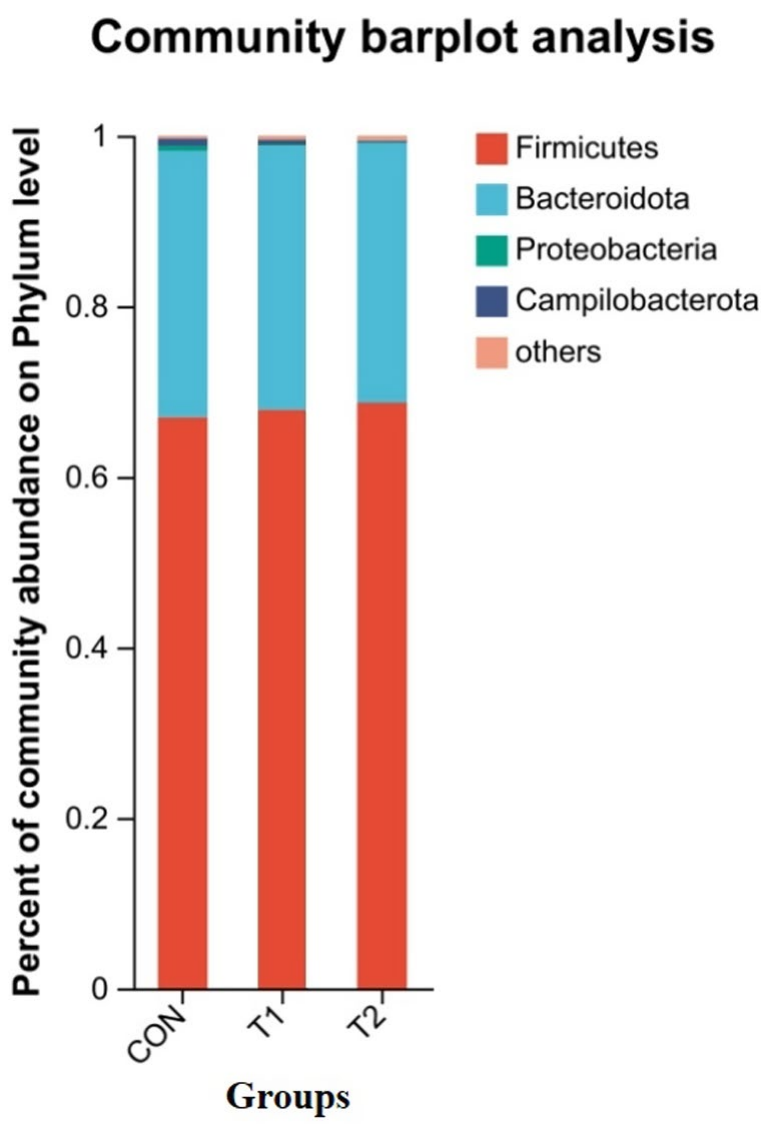
Effects of supplemental yeast culture on cecal microbes in broilers relative abundance at the phylum level %.

At the phylum level, the four most common phyla were *Firmicutes*, *Bacteroidota*, *Proteobacteria*, and *Campylobacterota* (Figure 2). *Firmicutes* was the most abundant phylum, followed by *Bacteroidota*, and small proportions of *Proteobacteria* and *Campylobacterota*, which accounted for over 95% of all phyla. As shown in Table 8, there was no significant difference in the relative abundance at the phylum level (*p* > 0.05).

**Table 8:** Effects of supplemental yeast culture on cecal microbes in broilers relative abundance at the phylum level %.

Item	CON	T1	T2	*p*-value
*Firmicutes*	66.99 ± 17.45	67.88 ± 9.15	68.68 ± 9.92	0.984
*Bacteroidota*	31.18 ± 17.07	29.81 ± 6.93	30.51 ± 10.21	0.891
*Proteobacteria*	0.67 ± 1.05	0.08 ± 0.03	0.10 ± 0.09	0.235
*Campylobacterota*	0.82 ± 1.28	0.63 ± 0.65	0.20 ± 0.23	0.473

Values within the same row with different lowercase superscript letters are significantly different (*p* < 0.05).

**Figure 3:**
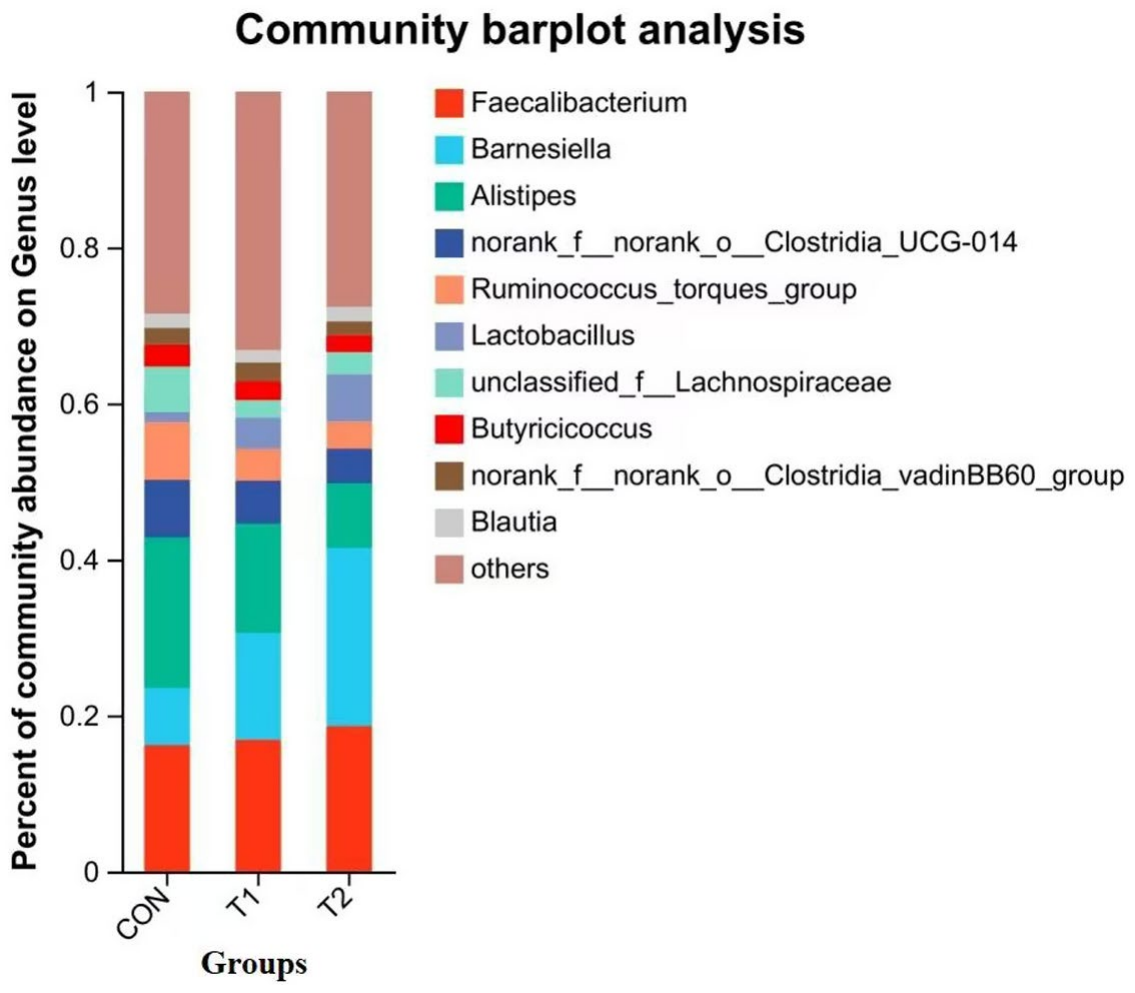
Effects of supplemental yeast culture on cecal microbes in broilers relative abundance at the genus level %.

At the genus level, the sequences from the 18 samples were categorized into 165 genera, of which the top 10 had a relative abundance of >1% (Figure 3). In addition, as listed in Table 9, the relative abundances of *Barnesiella* and *Lactobacillus* in the T2 group were significantly higher than those in the CON group (*p* < 0.05).

**Table 9:** Effects of supplemental yeast culture on cecal microbes in broilers relative abundance at the genus level %.

Item	CON	T1	T2	*p*-value
*Faecalibacterium*	16.21 ± 11.21	16.89 ± 4.01	18.63 ± 9.61	0.909
*Barnesiella*	7.33 ± 2.77^b^	13.69 ± 7.32	22.86 ± 7.01^a^	0.022
*Alistipes*	19.31 ± 22.69	13.98 ± 8.73	8.27 ± 3.54	0.477
*norank_f__norank_o__Clostridia_UCG-014*	7.38 ± 1.55	5.53 ± 2.89	4.45 ± 0.70	0.097
*Ruminococcus_torques_group*	7.37 ± 4.11	4.07 ± 2.38	3.46 ± 1.88	0.163
*Lactobacillus*	1.55 ± 0.98^b^	3.99 ± 3.14	7.37 ± 2.65^a^	0.014
*unclassified_f__Lachnospiraceae*	5.16 ± 1.91	2.30 ± 1.06	2.89 ± 1.37	0.086
*Butyricicoccus*	2.73 ± 0.48	2.36 ± 1.14	2.10 ± 1.65	0.675
*norank_f__norank_o__Clostridia_vadinBB60_group*	2.22 ± 2.12	2.41 ± 1.39	1.79 ± 0.69	0.711
*Blautia*	1.82 ± 1.02	1.63 ± 1.81	1.89 ± 1.17	0.966

Values within the same row with different lowercase superscript letters are significantly different (*p* < 0.05).

## 4 Discussion

Yeast and YC are feed ingredients, and research on their effects in poultry was initiated around 2000 (17). Various yeast products, including *Saccharomyces cerevisiae*-based preparations, had a positive impact on the body weight gain (BWG) and feed conversion ratio (FCR) in broilers (18, 19). Our study demonstrated that supplementation with YC in basal diets improved broiler growth performance, specifically, compared with the control group, ADG increased by approximately 5.5% (T1) and 4.0% (T2) over the 0–42 d period, while ADFI increased by approximately 4.2% (T1) and 3.3% (T2). In quantitative terms, YC supplementation at the T1 regimen increased ADG by 5.5% and improved CF digestibility by 26.5% during the starter phase, while reducing fecal indole and skatole by 6.7–7.2% and 4.8–6.0%, respectively. In agreement with our study, YC was found to have positive effects on the performance of broiler chicks (16). This observation could be attributed to the fact that YC contains functional nutrients, including amino acids, organic acids, and mannan, which may contribute to improved digestive function and nutrient utilization, although the underlying mechanisms were not directly investigated in the present study. Our results indicated that YC supplementation improved broiler growth performance, with the T1 group exhibiting the best performance.

Notably, although the T2 group received a consistently higher level of YC supplementation (0.625 kg/t) throughout the entire experimental period, the T1 group exhibited comparable growth performance to the T2 group across multiple indicators, and even outperformed the T2 group in terms of ADG (0–42 days) and F/G (21 days), suggesting the absence of a linear dose-dependent response. This phenomenon has also been reported in previous studies. Gao et al. (16) evaluated different supplementation levels of YC (0, 2.5, 5.0, and 7.5 g/kg) in broilers and found that growth performance was optimized at 2.5 g/kg, indicating the existence of an optimal supplemental dose rather than a simple linear dose–response relationship. Similarly, Zhen et al. (20) reported that optimal growth performance and feed efficiency in broilers were achieved at 0.8% YC supplementation. Several mechanisms may explain the comparable performance between T1 and T2 in the present study. First, the lower YC dosage (T1 regimen) may have been sufficient to exert beneficial effects on intestinal morphology and digestive enzyme activities, thereby achieving maximal biological responses. Second, the stepwise reduction in YC supplementation during the later growth phase (0.3125 kg/t in T1) may have better matched the physiological nutrient requirements during that period, whereas continuous high-dose supplementation did not confer additional growth advantages. Moreover, YC has been reported to enhance immune function and secretory IgA secretion, improvements that may reach a plateau even at relatively lower doses (16). From a production perspective, the T1 regimen (0.625 kg/t during the first 10 days followed by 0.3125 kg/t from days 11–42) may represent a more cost-effective supplementation strategy. Further studies investigating optimal YC levels for different growth phases would be of both theoretical and practical value.

The slaughter performance rate is an important indicator of the performance of broiler meat production. Our results showed that supplementation of YC to basal diets only significantly increased PBM in the T2 group. Fathi et al. (19) reported that YC had no significant effect on carcass traits such as carcass weight and leg muscle weight of broilers. Karaoglu and Durdag (21) found that supplementing broiler diets with *Saccharomyces cerevisiae* had no effect on hot or cold carcass percentage, and Chumpawadee et al. (22) found that including cassava yeast as a probiotic source had no influence. In contrast, Islam et al. (23) showed that adding YC to broiler diets significantly increased carcass, leg, and breast muscle weights. This finding differs from the results of the present study and could be ascribed to differences in yeast species or broiler breeds.

Previous studies have reported that YC supplementation does not result in significant differences in nutrient utilization between the control and treatment groups (24). In this study, nutrient utilization rates were enhanced in broilers. The addition of YC to the basal diet improved the utilization of DM, Ash, CF, and CP in broilers. The effects were particularly pronounced in the utilization of CF in the first phase, Ash and CF in the second phase, and CP in the third phase, with the T2 group showing the most significant improvement. This is consistent with Bradley and Savage (25), suggesting that supplementation with YC augments the feed utilization of animals. Previous studies have reported that YC supplementation may increase intestinal surface area and villus height in broilers (26), which could partly explain the improved nutrient digestibility observed in the present study. A combination of these factors may have contributed to higher nutrient digestibility in YC-fed broilers in this study.

Broiler excreta primarily contain skatole (3-methylindole) and indoles, which are the end products of L-tryptophan (L-try) degradation by various microorganisms. Several microbes can degrade L-Try to indole and indoleacetic acid, and some specific organisms can decarboxylate indoleacetic acid to form skatole (27). However, Hoque et al. (24) reported that YC supplementation did not result in significant differences in the emission of malodorous compounds between the control and treatment groups. In the present study, the addition of YC significantly decreased the levels of indole and skatole in excreta compared to the control group. These results suggest that YC supplementation may reduce fecal odor-related compounds. Yan et al. (28) mentioned that higher nutrient digestibility would result in reduced malodorous compounds emissions. Based on our experiment, this observation may be linked to the addition of YC, which improves nutrient utilization and increases the abundance of beneficial bacteria.

The fundamental nutrients for muscles are proteins, fat, water, and ash. The nutrient content of muscle is crucial for meat quality. Inosine monophosphate (IMP) is a flavor-enhancing substance that plays a crucial role in the umami taste of the muscle, making it a key indicator of this taste (29). In this study, IMP levels were numerically higher in the T2 group, but the difference was not statistically significant. Li et al. (30) showed that the addition of antibiotics resulted in a significant increase in abdominal fat weight, abdominal fat percentage, and triglyceride and cholesterol levels in the blood. In addition, Zhou et al. (31) reported that the ability of nuciferine could reduce fat deposition in broiler chickens through regulation of lipid metabolism and TG/TC balance. In this study, YC supplementation significantly reduced TG and TC contents. These findings suggest that YC may reduce muscle lipid deposition, potentially through modulation of lipid metabolism pathways, with the T2 group showing the most significant effect. Active dry yeast and YC exerted an effect on milk fat percentage and protein content. This finding suggests that yeast preparations can affect fat and protein metabolism in ruminants, thereby influencing product quality (32, 33). Our study further found that YC supplementation did not significantly affect the levels of crude protein and crude fat in muscle. In summary, YC supplementation mainly reduced lipid-related parameters (TG and TC), with no significant effects on other muscle nutrient components.

Our study demonstrated that the addition of YC to the basal diet did not alter the alpha and beta diversity of cecal microbiota in broilers, indicating that YC supplementation resulted in similar cecal microbial community structure. The microbiota analysis was based on six samples per treatment group, which may have limited the statistical power to detect subtle differences in microbial community composition. Therefore, microbiota-related findings should be interpreted with caution. The intestinal microbiota represents the largest and most complex microbial ecosystem in the body. Studies have demonstrated that YC may influence specific bacterial taxa without significantly changing the overall microbial diversity observed in this study. In livestock and poultry, promoting the growth of beneficial bacteria in the intestinal tract and inhibiting the growth of harmful ones are important functions of feed additives (34, 35). The present study found that the dominant phyla were *Firmicutes* and *Bacteroidetes*. Wu et al. (36) reported that *Firmicutes* exhibited the highest relative abundance among the cecum microorganisms of broilers. In this study, too, comparable results were obtained. *Bacteroidetes* aid the host in absorbing and utilizing polysaccharides efficiently (37), and *Firmicutes* primarily work with *Bacteroidetes* to help in the digestion of certain substances that cannot be acted upon by the host (38). In this study, although no significant differences were observed at the phylum level, numerical changes in the relative abundances of several phyla were observed following YC supplementation. However, these differences were not statistically significant. *Proteobacteria* include many pathogens, and an increase in their relative abundance will not only disturb the balance of the microbial community structure but also enhance the probability of disease (39). Similarly, a decreasing trend in the relative abundance of *Proteobacteria* and *Campylobacterota* was observed, although the differences were not statistically significant. It is therefore possible that YC supplementation selectively modulates specific bacterial taxa rather than substantially altering the overall microbial community structure.

The microorganisms were further analyzed at the genus level. After the addition of YC, the T2 group showed significantly increased abundances of *Barnesiella* and *Lactobacillus* compared with the control group. *Barnesiella* belongs to *Bacteroidetes* and is a newly identified genus. Furthermore, as a valuable “antitumor probiotic” in immunomodulation, *Barnesiella intestinihominis* accumulates in the colon and increases the number of intratumoral interferon-γ-producing γδT cell (40). Markazi et al. (41) found that whole yeast products increased cecal *Lactobacillus* in laying hens. *Lactobacillus* is beneficial in maintaining the balance of intestinal microbiota and promotes digestion in host nutrition (42). The observed increases in *Barnesiella* and *Lactobacillus* suggest that YC supplementation may selectively influence specific bacterial genera associated with intestinal health. Whether these microbial changes contribute directly to the improvements in growth performance and nutrient digestibility requires further investigation.

Although several responses reached statistical significance, the magnitude of improvement was relatively modest. Nevertheless, even modest improvements in feed efficiency and nutrient utilization may translate into meaningful economic benefits under large-scale commercial production conditions. Since no economic analysis was conducted in the present study, further research is required to evaluate the cost-effectiveness of YC supplementation under practical production conditions.

## 5 Limitations

Despite the promising findings, several limitations should be acknowledged. First, this study was conducted at a single research location, which may limit the generalizability of the results to other production settings. Second, only one commercial yeast culture product was evaluated, so the observed effects may not represent all yeast culture formulations. Third, intestinal morphology was not assessed, and immune-response indicators were not measured, which restricts the mechanistic interpretation of growth performance improvements. Fourth, the microbiota analysis was based on a limited sample size (*n* = 6 per treatment) and was performed at a single time point, which may have reduced the statistical power and temporal resolution of microbiota dynamics. Finally, no economic or cost–benefit analysis was conducted, which limits the practical evaluation of dietary yeast culture supplementation under commercial production conditions. Future studies should address these limitations to provide a more comprehensive understanding of the effects of yeast culture in broilers.

## 6 Conclusion

Dietary supplementation with YC positively affected broiler performance by improving growth performance and nutrient utilization, reducing fecal odor-related compounds, and decreasing muscle triglyceride and total cholesterol contents. YC supplementation did not markedly alter the overall diversity of the cecal microbiota but selectively increased the relative abundances of *Lactobacillus* and *Barnesiella*. These findings suggest that the beneficial effects of YC on broiler production may be associated with improvements in intestinal function and selective modulation of the gut microbiota. Overall, YC represents a promising feed additive for enhancing broiler health and production performance.

## Data Availability

The data presented in the study are deposited in the Genbank repository, accession number PRJNA1504459.
